# Altered Function and Expression of ABC Transporters at the Blood–Brain Barrier and Increased Brain Distribution of Phenobarbital in Acute Liver Failure Mice

**DOI:** 10.3389/fphar.2018.00190

**Published:** 2018-03-06

**Authors:** Li Liu, Mingxing Miao, Yang Chen, Zhongjian Wang, Binbin Sun, Xiaodong Liu

**Affiliations:** Center of Drug Metabolism and Pharmacokinetics, School of Pharmacy, China Pharmaceutical University, Nanjing, China

**Keywords:** blood–brain barrier, acute liver failure, P-glycoprotein, ABC efflux transporters, phenobarbital

## Abstract

This study investigated alterations in the function and expression of P-glycoprotein (P-GP), breast cancer resistance protein (BCRP), and multidrug resistance-associated protein 2 (MRP2) at the blood–brain barrier (BBB) of acute liver failure (ALF) mice and its clinical significance. ALF mice were developed using intraperitoneal injection of thioacetamide. P-GP, BCRP, and MRP2 functions were determined by measuring the ratios of brain-to-plasma concentration of rhodamine 123, prazosin, and dinitrophenyl-*S*-glutathione, respectively. The mRNA and proteins expression levels of P-GP, BCRP, and MRP2 were evaluated with quantitative real-time PCR and western blot, respectively. MDCK-MDR1 and HCMEC/D3 cells were used to document the effects of the abnormally altered components in serum of ALF mice on the function and expression of P-GP. The clinical significance of alteration in P-GP function and expression was investigated by determining the distribution of the P-GP substrate phenobarbital (60 mg/kg, intravenous administration) in the brain and loss of righting reflex (LORR) induced by the drug (100 mg/kg). The results showed that ALF significantly downregulated the function and expression of both P-GP and BCRP, but increased the function and expression of MRP2 in the brain of mice. Cell study showed that increased chenodeoxycholic acid may be a reason behind the downregulated P-GP function and expression. Compared with control mice, ALF mice showed a significantly higher brain concentration of phenobarbital and higher brain-to-plasma concentration ratios. In accordance, ALF mice showed a significantly larger duration of LORR and shorter latency time of LORR by phenobarbital, inferring the enhanced pharmacological effect of phenobarbital on the central nervous system (CNS). In conclusion, the function and expression of P-GP and BCRP decreased, while the function and expression of MRP2 increased in the brain of ALF mice. The attenuated function and expression of P-GP at the BBB might enhance phenobarbital distribution in the brain and increase phenobarbital efficacy on the CNS of ALF mice.

## Introduction

Acute liver failure (ALF) is often associated with serious neurological complications and is responsible for mortality such as encephalopathy, brain edema, and hepatic coma. This is likely due to the accumulation of disordered neuroactive or neurotoxic components in the brain (Villano et al., 2012; Butterworth, 2015; Hadjihambi et al., 2017). Cerebral complications during ALF status may be attributed to many factors including increases in the permeability of the blood–brain barrier (BBB), vasogenic edema, and intracranial hypertension (Cauli et al., 2011; Scott et al., 2013).

Under the physiological state, the BBB forms a physical and biochemical barrier to maintain brain homeostasis via effluxing endogenous and exogenous substances from the brain to peripheral blood and restricting the entry of harmful substances into the central nervous system (CNS). Some ATP-binding cassette (ABC) efflux transporters including P-glycoprotein (P-GP/Abcb1), breast cancer resistance protein (BCRP/Abcg2), and multidrug resistance-associated protein 2 (MRP2/Abcc2) have been identified at the BBB and demonstrated important roles in CNS homeostasis by extruding harmful substances. Growing evidence has demonstrated that some diseases such as liver failure (Nguyen, 2012; Jin et al., 2013), diabetes (Liu and Liu, 2014), stroke (Yang and Rosenberg, 2011), and epilepsy (Jing et al., 2010; Yao et al., 2012) impair BBB function, leading to a disruption of CNS homeostasis. The alterations in the function and expression of these ABC efflux transporters are important factors contributing to BBB dysfunction under a disease status (Jing et al., 2010; Yao et al., 2012; Jin et al., 2013; Liu and Liu, 2014).

We once reported that both acute and chronic liver failure induced by thioacetamide (TAA) downregulated P-GP function and expression, and upregulated MRP2 function and expression at the BBB of rats (Jin et al., 2013). Similarly, both function and expression of BCRP at the BBB of rats were decreased by ALF or bile duct ligation, leading to increases in brain distribution of prazosin (Li et al., 2016; Xu et al., 2016). These results indicated that the altered function of ABC transporters at the BBB by ALF might affect the brain distribution, CNS activity, and toxicity of their substrates. Phenobarbital, an antiepileptic drug, was reported to be a substrate for P-GP (Yang and Liu, 2008; Mairinger et al., 2012). It was also reported that diabetes significantly down-regulated the function and expression of P-GP at the BBB of mice, leading to increases in brain distribution of phenobarbital and its efficacy/toxicity (Liu et al., 2007).

The aims of this study are: (1) to simultaneously demonstrate the function and expression of P-GP, BCRP, and MRP2 at the BBB of TAA-induced ALF mice; (2) to document the effects of the components found to be abnormally altered in the serum of ALF mice on P-GP function and expression using HCMEC/D3 and MDCK-MDR1 cells; and (3) to investigate the effects of altered P-GP function and expression at the BBB of ALF mice on brain distribution and CNS activity/toxicity of phenobarbital. The CNS efficacies of phenobarbital were indexed as both loss of righting reflex (LORR) and the latency time. The results are expected to highlight the clinical significance of alteration in function and expression of ABC transporters at the BBB by ALF.

## Materials and Methods

### Reagents

Thioacetamide, rhodamine 123, and Evans blue were purchased from Sigma-Aldrich (St. Louis, MO, United States). *S*-(2,4-dinitrophenyl)-glutathione (DNP-SG) and 1-chloro-2,4-dinitrobenzene were supplied by Toronto Research Chemicals (North York, ON, Canada). Phenobarbital, prazosin, midazolam, and 1-hydroxymidazolam were purchased from National Institutes for Food and Drug Control (Beijing, China). M-MLV reverse transcriptase, TRIzol reagent, and SYBR Green Real-Time (RT) PCR Master Mix were purchased from ThermoFisher (Carlsbad, CA, United States). The kits for the analysis of ammonia, glutathione (GSH), total bilirubin, and malondialdehyde (MDA), and the activity for superoxide dismutases (SOD), catalase (CAT), alkaline phosphatase (ALP), aspartate amino transferase (AST), and alanine amino transferase (ALT) were all from Nanjing Jiancheng Bioengineering Institute (Nanjing, China). Radioimmunoprecipitation (RIPA) lysis buffer, assay kit for nitric oxide (NO), tumor necrosis factor-alpha (TNF-α), and bicinchoninic acid (BCA) kit for protein content were supplied by Beyotime Institute of Biotechnology (Shanghai, China). Pure water was prepared by a Milli-Q system (Millipore, Bedford, MA, United States). Other chemicals were all analytical grade and commercially available.

### Animals

Male C57BL/6 mice were provided by Sino-British Sipper & BK Lab Animal Co., Ltd. (Shanghai, China). Animals were housed under specific pathogen-free environment under constant temperature (25–28°C) and humidity (50–60%), with 12 h light/dark cycle. Rodent chow and water were autoclaved and provided *ad libitum*. All animals were acclimated to the environment for 5 days before the experiments. Animal experiments were carried out according to the Institutional Guidelines for the Care and Use of Laboratory Animals and were authorized by the Animal Ethics Committee of China Pharmaceutical University.

### Induction of ALF Mice

A well-verified model of TAA-induced ALF was used. Briefly, male C57BL/6 mice, aged 12–14 weeks, were intraperitoneally injected TAA (300 mg/kg) once daily for 3 days to induce ALF (Gomides et al., 2014). Drinking water containing 5% dextrose, 0.149% KCl, and 0.45% NaCl (v/v) was given to the mice in order to minimize hypoglycemia and electrolytes imbalance (Farjam et al., 2012). Control mice were given with physiological saline instead of TAA. All experiments were conducted at 6 h following the last administration of TAA or vehicle.

### Physiological and Biochemical Parameters

Liver weights and serum parameters were measured to validate ALF development. AST, ALT, ALP, ammonia, total bilirubin, and TNF-α levels in the serum were measured using commercial kits according to manufacturer’s instructions. NO, GSH, CAT, SOD, and MDA levels in the brain were determined using commercial kits according to manufacturer’s instructions.

### Liver Histology

Livers from control and ALF mice were partly excised and sliced into prisms between 0.3 and 0.5 cm, immobilized in 10% formalin solution. The prisms were embedded in paraffin after dehydration with alcohol. Liver specimens (4 μm) were cut and stained with hematoxylin–eosin (H&E), and subsequently measured by light microscopy.

### Brain Distribution of Rhodamine 123, Prazosin, and DNP-SG in Mice

Brain distributions of rhodamine 123 (a substrate of P-GP), prazosin (a substrate of BCRP), and DNP-SG (a substrate of MRP2) were determined according to a previous method (Jin et al., 2013). Briefly, mice were intravenously given rhodamine 123 (0.8 mg/kg), prazosin (2 mg/kg), or 1-chloro-2,4-dinitrobenzene (precursor of DNP-SG, 10 mg/kg). At 15, 30, and 45 min following intravenous administration of respective P-GP, BCRP, and MRP2 substrates, the mice were sacrificed under light ether anesthesia, and brain, liver, and blood samples were immediately collected. Serum and plasma samples were prepared for determining biochemical parameters and substrate concentrations, respectively. Hepatic microsomes were prepared for the study of Cyp3a11 activity and expression. The brain tissues were separated for assessing substrate levels, biochemical parameters, and expression of the three ABC efflux transporters. The concentrations of rhodamine 123, prazosin, and DNP-SG in the plasma and brain were determined with according to the HPLC methods previously described (Jin et al., 2013). To evaluate the effect of ALF on BBB integrity, control and ALF mice were sacrificed under light ether anesthesia 1 h following intravenous injection of Evans blue (0.1 mL/10 g weight). Evans blue levels in brain were measured using spectrofluorophotometry (Liu et al., 2007).

### Effects of Abnormally Altered Components on P-GP Function and Expression in HCMEC/D3 and MDCK-MDR1 Cells

The effects of unconjugated bilirubin (UCB), cholic acid (CA), deoxycholic acid (DCA), lithocholic acid (LCA), chenodeoxycholic acid (CDCA), and ursodeoxycholic acid (UDCA) on P-GP function and expression were investigated in HCMEC/D3 and MDCK-BCRP (MDCK type II cells transfected with human BCRP) cells. In brief, sub-confluent (80%) cells were incubated with medium containing different concentrations of UCB (0, 10, or 25 μM) and bile acids (0, 10, or 100 μM) for 24 h. The concentrations of UCB and individual bile acids were chosen based on previous studies (Komori et al., 2014; Liu et al., 2014; Xu et al., 2016). It is noted that the data from a MTT study demonstrated that cell viability was not impaired by these agents at the tested concentrations (**Figures 2A,B**). The function and expression of P-GP in HCMEC/D3 and MDCK-MDR1 cells were evaluated using rhodamine 123 (0.1 μg/mL) and vincristine (3 μg/mL) uptake and western blotting, respectively.

### Evaluation of Phenobarbital Transport in MDCK-MDR1 and MDCK-BCRP Cells

MDCK type II cells transfected with human P-GP (MDCK-MDR1), BCRP (MDCK-BCRP), and the MDCKII wild-type cells were seeded on transparent polyester membrane filters (Transwell^®^, 6.5 mm diameter, 0.4 μm pore size; Corning Costar Co., Cambridge, MA, United States) at a density of 4 × 10^5^ cells/cm^2^, cultured, and used for transport assays after reaching confluence. The phenobarbital transport study was carried out in MDCK-MDR1, MDCK-BCRP, and respective MDCKII wild-type cells based on the previously described methods (Luna-Tortós et al., 2008, 2010). The volumes of lower and upper transport compartment were 0.6 and 0.4 mL, respectively. The initial concentration of phenobarbital was 10 μM in donor compartment. The functions of P-GP and BCRP on the apical membrane of transfected MDCK cell monolayers were verified using transport of rhodamine 123 and prazosin, respectively.

Permeabilities from apical-to-basal (*P*_appAP-BL_) and basal-to-apical (*P*_appBL-AP_) were calculated using the equation: *P*_app_ (nm/s) = (d*Q*/d*t*)/(*A* × *C*_0_ × 60), where d*Q*/d*t* (μg/min), *A*, and *C*_0_ are the permeability rate of the drug, surface area of the monolayer, and initial drug concentration in the donor chamber, respectively. Transport ratio (TR) was obtained by dividing *P*_app,BL-AP_ by *P*_app,AP-BL_. Corrected transport ratios (cTRs) were the ratios of TR in MDR1 or BCRP-transfected cells to that in the wild-type cells (Schwab et al., 2003). A cTR value >1.5 suggests the existence of active and asymmetrical transport (Schwab et al., 2003).

### Effect of ALF on the Duration of Phenobarbital-Induced LORR and the Latency Time of LORR in Mice

Duration of LORR and its latency time were indexed as pharmacodynamic endpoint of phenobarbital. ALF mice and normal control mice were intravenously administrated with 100 mg/kg of phenobarbital. Then the mice were cared in the supine posture in a V-shaped plastic box when the mice become ataxic and the time periods mentioned above were recorded (Liu et al., 2007; Wang et al., 2012).

### Effect of ALF on Phenobarbital Exposure in Plasma and Brain of Mice

Acute liver failure and normal control mice were randomized into two groups (*n* = 12). The mice were administrated with 60 mg/kg of phenobarbital through tail vein. Then the mice were sacrificed via decapitation under light ether anesthesia and the brain or blood samples were obtained at 0.17, 0.5, 1, 2, 6, 12, and 24 h. Blood samples were collected in heparinized tubes and plasma samples were obtained by centrifuging. The brain and plasma concentrations of phenobarbital were determined using an established HPLC method (Liu et al., 2007).

### Hepatic Microsomes Preparation and Cyp3a11 Activity Measurement

Hepatic microsomes were obtained freshly from ALF and control mice based on a literature (Liu et al., 2012). The microsomes were used for mouse Cyp3a11 activity and protein analysis.

Cyp3a11 activity of liver microsomes was determined based on the production of the metabolite 1-hydroxymidazolam from the substrate midazolam (Chavan et al., 2015). In brief, midazolam (5 μM) was incubated at 37°C with hepatic microsomes (0.2 mg/mL) and an NADPH generating system (final volume of 200 μL) for 10 min. The reaction was terminated by adding 200 μL of ice-cold acetonitrile. The amount of 1-hydroxymidazolam formed after incubation was measured using an HPLC method (Jia et al., 2014).

### QRT-PCR Analysis

The mRNA levels of Abcb1a/1b, Abcc2, and Abcg2 in the brain and Cyp3a11 in the liver of experimental mice were determined by QRT-PCR. Total RNAs were extracted from frozen brain and liver using Trizol and used as the template for cDNA synthesis using cDNA Reverse Transcription Kit (Toyobo, Tokyo, Japan). RT-PCR was performed on an ABI 7500 Fast RT-PCR System (Applied Biosystems, Foster City, CA, United States) for relative quantification. PCR primer sequences (Yingjun Biotech, Shanghai, China) are shown in **Table 1**. Relative mRNA expression levels were determined after normalizing the expression levels by β-actin expressions (2^-ΔΔ*C*_t_^ method).

**Table 1:** Primer characteristics used for QRT-PCR analysis.

Gene	Accession no.	Forward primer	Reverse primer
Abcb1a	NM_011076.2	5′-GCAGGTTGGCTAGACAGGTTGT-3′	5′-GAGCGCCACTCCATGGATAA-3′
Abcb1b	NM_011075.2	5′-GCTGGACAAGCTGTGCATGA-3′	5′-TGGCAGAATACTGGCTTCTGCT-3′
Abcc2	NM_013806.2	5′-GGATAATGAGGCGCCGTGGGT-3′	5′-CCGGCCGATACCGCACTTGA-3′
Abcg2	NM_011920.3	5′-AATCAGGGCATCGAACTGTCA-3′	5′-CAGGTAGGCAATTGTGAGGAAGA-3′
Cyp3a11	NM_007818.3	5′-GGATGAGATCGATGAGGCTCTG-3′	5′-CAGGTATTCCATCTCCATCACAGT-3′
β-Actin	NM_007393.5	5′-CCACTGCCGCATCCTCTTCC-3′	5′-CTCGTTGCCAATAGTGATGACCTG-3′


### Western Blot Analysis

The protein expression levels of P-GP, BCRP, and MRP2 in the cells and mouse brain, and Cyp3a11 in mouse liver microsomes were determined by western blot analysis. At first, the tissue or cell samples were homogenized and lysed in RIPA lysis buffer solution. The protein concentrations were determined by the BCA protein quantitative kit. Equal amounts of proteins were subjected to sodium dodecyl sulfate–polyacrylamide gel electrophoresis (SDS–PAGE) and transferred to PVDF membranes. Blots were blocked for 2 h in 5% nonfat dry milk–TBS–0.1% Tween 20 and then washed. Thereafter, the membranes were incubated overnight at 4°C with primary antibodies against P-GP (0.5 μg/mL, Merck-Millipore Co., Seattle, WA, United States), MRP2 (0.5 μg/mL, Abcam Co., Cambridge, United Kingdom), BCRP (0.5 μg/mL, Abcam Co., Cambridge, United Kingdom), Cyp3a11 (0.25 μg/mL, Santa Cruz Biotechnology Co., Santa Cruz, CA, United States), and β-actin (0.2 μg/mL, Abcam Co., Cambridge, United Kingdom) according to the manufacturer’s instructions. Afterward, membranes were incubated for 2 h at room temperature with a horseradish peroxidase-conjugated secondary antibody (0.2 μg/mL, Abcam Co., Cambridge, United Kingdom). The immunoreactivity was detected using Super Signal West Femto Chemiluminescent Substrate (Thermo Fisher Scientific Inc., Rockford, IL, United States) by a gel imaging system (Bio-Rad ChemiDoc XRS+ System, Hercules, CA, United States). Relative expressions of target proteins were normalized by the reference bands of β-actin.

### Statistical Analysis

All results were expressed as mean ± standard deviation (SD). Differences between two groups were determined by Student’s *t*-test. A *P-*value <0.05 denoted a significant difference.

## Results

### Alterations of Physiological and Biochemical Parameters in ALF Mice

Thioacetamide-induced ALF mice were confirmed by physiological and biochemical parameters (**Table 2**). Elevated ALT and AST levels are biomarkers of liver failure. Compared to normal control mice, a fivefold increase in ALT and a 13-fold increase in AST were found in the serum of ALF mice. In addition to the raised levels of ALT and AST, serum ALP, total bilirubin, and TNF-α of ALF mice also significantly increased compared to normal mice. However, serum ammonia levels were not altered by ALF.

**Table 2:** Physiological and biochemical parameters of the ALF and control mice.

Parameters	Control (*n* = 6)	ALF (*n* = 6)
Liver weight/body weight (%)	3.1 ± 0.1	4.5 ± 0.2*
ALT (units/L)	38.6 ± 10.7	192.4 ± 33.1**
AST (units/L)	26.4 ± 8.6	349.2 ± 72.4**
ALP (units/L)	246.4 ± 33.7	619.4 ± 118.1**
Total bilirubin (μmol/L)	5.4 ± 1.4	24.6 ± 6.6**
Conjugated bilirubin (μmol/L)	3.1 ± 0.8	15.5 ± 4.3**
Total bile acids (μmol/L)	32.7 ± 9.6	113.4 ± 27.9**
Ammonia (μmol/L)	76.7 ± 14.2	86.1 ± 17.8
TNF-α (pg/mL)	6.7 ± 2.5	47.5 ± 10.7**
NO (brain) (μM/g)	13.5 ± 4.0	12.1 ± 3.9
GSH (brain) (μM/g)	3.5 ± 1.4	1.8 ± 0.8*
MDA (brain) (mmol/g)	33.7 ± 7.2	75.3 ± 15.3**
SOD (brain) (units/mg protein)	7.7 ± 2.2	5.9 ± 2.8
CAT (brain) (units/mg protein)	18.4 ± 4.0	11.4 ± 4.1**


*Data are expressed as mean ± SD (*n* = 6). ^∗^*P* < 0.05, ^∗∗^*P* < 0.01 vs. control.*

Nitric oxide, GSH, MDA, SOD, and CAT levels (**Table 2**) in the brain of mice were also determined to indicate oxidative stress. The NO and SOD levels in the brain did not show any difference among the groups. However, the levels of GSH and CAT were significantly decreased, while the levels of MDA were increased in the brain of ALF mice compared with those in control mice, which indicated that oxidative stress had occurred in the brain of ALF mice.

### Liver Histology

Histopathological assessment was also conducted to confirm the liver injury induced by ALF. Livers from ALF mice showed clearly hydropic degeneration, inflammatory infiltration, and hepatocellular damage. The results of histopathological analysis and biochemical parameters confirmed the impaired liver function of the mice after TAA administration.

### Effect of ALF on the Distribution of Rhodamine 123, Prazosin, and DNP-SG in the Brain

This experiment was designed to determine whether ALF affected P-GP, BCRP, and MRP2 function at the BBB of mice. The levels of rhodamine 123, prazosin, and DNP-SG in plasma and brain were measured to assess the function of P-GP, BCRP, and MRP2 at the BBB of mice, respectively (**Table 3**). The results showed that the plasma concentration of rhodamine 123 was significantly increased in ALF mice, contrarily, plasma concentration of prazosin was significantly decreased in ALF mice compared with control mice. However, ALF did not alter plasma concentration of DNP-SG. Since substrate concentration in the brain can be affected by its plasma concentration, brain-to-plasma concentration ratios of rhodamine 123, prazosin, and DNP-SG were calculated to reflect brain penetration (Jin et al., 2013). It was found that the brain-to-plasma ratios of rhodamine 123 and prazosin were significantly increased, while the brain-to-plasma ratios of DNP-SG were significantly decreased in ALF mice compared with control mice. These results indicated that ALF down-regulated P-GP and BCRP function but up-regulated MRP2 function at the BBB of mice.

**Table 3:** Effect of ALF on rhodamine 123, DNP-SG, and prazosin distribution in plasma and brain.

	Control (*n* = 6)	ALF (*n* = 6)
**Rhodamine 123**		
Plasma level (ng/mL)	17.72 ± 3.43	22.31 ± 3.40*
Brain level (ng/g)	2.65 ± 0.70	5.20 ± 1.32**
Brain-to-plasma ratio (mL/g)	0.15 ± 0.02	0.23 ± 0.03**
**DNP-SG**		
Plasma level (μg/mL)	9.22 ± 2.63	11.81 ± 3.13
Brain level (μg/g)	2.26 ± 0.71	1.37 ± 0.34*
Brain-to-plasma ratio (mL/g)	0.25 ± 0.04	0.12 ± 0.02**
**Prazosin**		
Plasma level (ng/mL)	188.32 ± 32.66	146.05 ± 29.86*
Brain level (ng/g)	31.84 ± 4.97	41.96 ± 9.81*
Brain-to-plasma ratio (mL/g)	0.17 ± 0.02	0.29 ± 0.03**
**Evens blue**		
Brain level (ng/mg)	2.35 ± 0.31	2.74 ± 0.46


*Data are expressed as mean ± SD (*n* = 6). ^∗^*P* < 0.05, ^∗∗^*P* < 0.01 vs. control mice.*

To investigate whether the altered brain concentrations of substrates resulted from integrity alteration of the BBB, the brain concentration of Evans blue was determined at 1 h following its intravenous administration. No significant differences of the concentrations between ALF mice and control mice were found, indicating that the integrity of the BBB was not affected by ALF.

### Effect of ALF on mRNA Levels of Abcb1, Abcg2, and Abcc2 in Mouse Brain

**FIGURE 1:**
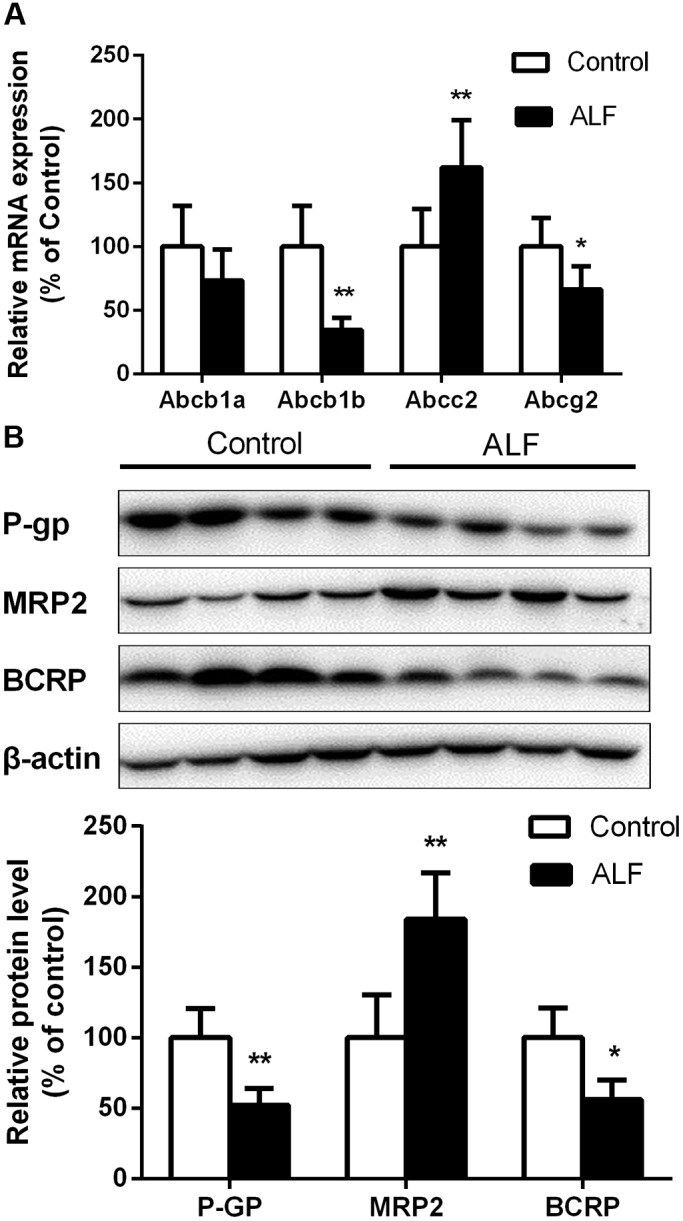
The mRNA **(A)** and protein **(B)** level of P-GP, MRP2, and BCRP in brain of control (white bar) and ALF (shaded bar) mice. Ratios of relative staining intensity for P-GP, MRP2, and BCRP in control and ALF mouse brain are described in **(B)**. Data are expressed as mean ± SD (*n* = 4). ^∗^*P* < 0.05, ^∗∗^*P* < 0.01 vs. control mice.

The mRNA levels of P-GP (Abcb1), BCRP (Abcg2), and MRP2 (Abcc2) in the brain were measured using QRT-PCR (**Figure 1A**). The results showed that Abcb1b mRNA levels in the brain of ALF mice were significantly reduced to 34% of those in control mice. Brain Abcb1a mRNA levels had a decreasing trend in ALF mice, but was without statistical difference. Meanwhile, BCRP mRNA levels in the brain of ALF mice were markedly attenuated to 67% of control mice. However, ALF significantly enhanced Abcc2 mRNA levels in the brain, which was a 1.6-fold increase compared with normal mice.

### Effect of ALF on Protein Levels of P-GP, BCRP, and MRP2 in Mouse Brain

Protein levels of P-GP, BCRP, and MRP2 in mouse brain were determined by western blot analysis (**Figure 1B**). It was consistent with the decreases in Abcb1a/1b and Abcg2 mRNA levels that ALF significantly decreased levels of P-GP and BCRP proteins brain of mice, whose protein levels were reduced to 52% and 56% of control mice. On the contrary, the expression of MRP2 protein in the brain of ALF mice was significantly increased to 184% of control mice.

### Effects of Abnormally Altered Components on P-GP Function and Expression in HCMEC/D3 and MDCK-MDR1 Cells

**FIGURE 2:**
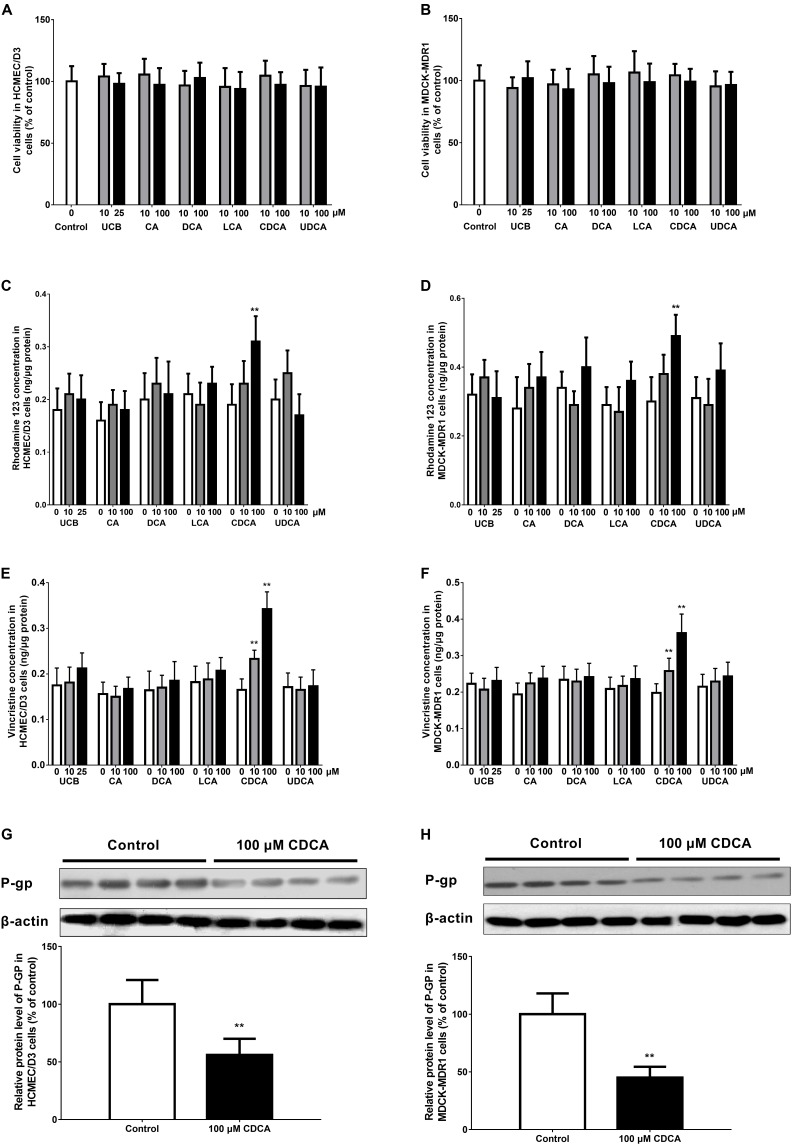
Effects of abnormally altered components (UCB, unconjugated bilirubin; CA, cholic acid; DCA, deoxycholic acid; LCA, lithocholic acid; CDCA, chenodeoxycholic acid; and UDCA, ursodeoxycholic acid) in ALF mouse serum on the function and expression of P-GP in HCMEC/D3 and MDCK-MDR1 cells, respectively. Cell viability **(A,B)**, rhodamine 123 uptake **(C,D)**, vincristine uptake **(E,F)**, and P-GP expression **(G,H)** were determined in HCMEC/D3 and MDCK-MDR1 cells after treatment with abnormally altered components for 24 h, respectively. Data are expressed as mean ± SD (*n* = 4). ^∗^*P* < 0.05, ^∗∗^*P* < 0.01 vs. control.

The present data indicated that ALF mice exhibited significant increases in serum levels of UCB and bile acids. Thus, the effects of these abnormally altered components on P-GP function and expression in both HCMEC/D3 and MDCK-MDR1 cells were investigated. The uptake of rhodamine 123 and vincristine was significantly increased with 100 μM CDCA in the HCMEC/D3 and MDCK-MDR1 cells, respectively (**Figures 2C–F**). Meanwhile, the protein expression of P-GP was also down-regulated with 100 μM CDCA in the HCMEC/D3 and MDCK-MDR1 cells, respectively (**Figures 2G,H**). However, all the other tested bile acids (UCB, CA, DCA, LCA, and UDCA) did not affect the function of P-GP in the HCMEC/D3 and MDCK-BCRP cells (**Figures 2C–F**). These results indicated that the raised CDCA in serum might decrease the function and expression of P-GP at the BBB of ALF mice.

### Transport of Phenobarbital by MDCK-MDR1 and MDCK-BCRP Cells

**FIGURE 3:**
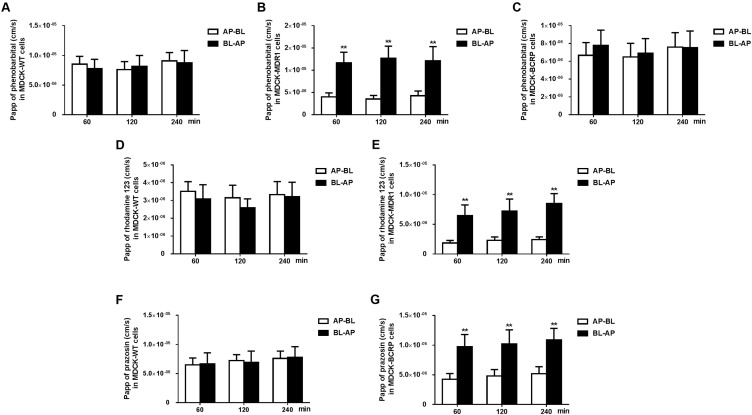
The apical–basolateral (AP–BL) and basolateral–apical (BL–AP) apparent permeability coefficients (Papp) of phenobarbital across MDCK type II cells transfected with human P-GP (MDCK-MDR1) **(B)**, BCRP (MDCK-BCRP) **(C)**, and respective MDCKII wild-type (MDCK-WT) **(A)** cells. Papp of reference substrate rhodamine 123 (P-GP) was tested in MDCK-WT **(D)** and MDCK-MDR1 **(E)** cell monolayers. Papp of reference substrate prazosin (BCRP) was tested in MDCK-WT **(F)** and MDCK-BCRP **(G)** cell monolayers. Phenobarbital, rhodamine 123, and prazosin concentrations were set to be 10 μM, respectively. Data are expressed as mean ± SD (*n* = 4). ^∗∗^*P* < 0.01 vs. Papp_AP-BL_.

Rhodamine 123 and prazosin are commonly used as a positive control in transcellular transport assays in P-GP or BCRP overexpressing cells, respectively. The P-GP substrate rhodamine 123 and BCRP substrate prazosin showed directional transport (basolateral to apical) with cTR values of 3.64 and 2.05 in MDCK-MDR1 and MDCR-BCRP cells (**Figures 3D–G**), respectively. Phenobarbital also showed asymmetrical transport (basolateral to apical) with cTR values of 2.94 in MDCK-MDR1 cells (**Figures 3A,B**), but not in MDCR-BCRP cells (**Figures 3A,C**). This result indicated that phenobarbital was a substrate of P-GP, which is in agreement with a previous study (Luna-Tortós et al., 2008).

### Effect of Phenobarbital on the Duration of LORR in ALF and Normal Control Mice

Both ALF and control mice exhibited LORR following intravenous administration of phenobarbital (100 mg/kg), and the agent showed a stronger effect in ALF mice (**Table 4**). The latency and duration time of LORR in control mice were 51.8 ± 14.6 and 68.2 ± 14.9 min, respectively. Nevertheless, the latency time of LORR in ALF mice was only 26.4 ± 7.8 min, which was significantly shorter than that in control mice. Five ALF mice fell into a coma and died after 10 h following intravenous administration of phenobarbital. Only 58% of ALF mice were recorded over the duration time, which was prolonged to 113.6 ± 20.2 min. The results demonstrated that the pharmacological and toxicological effect of phenobarbital on CNS was strengthened in ALF mice.

**Table 4:** Effect of ALF on the latency and duration time of the phenobarbital-induced loss of the righting reflex (LORR) in normal control mice and ALF mice.

Group	*n*	Latency time	Duration time
		of LORR (min)	of LORR (min)
Control (PB 100 mg/kg)	12	51.8 ± 14.6	68.2 ± 14.9
ALF (PB 100 mg/kg)	12	26.4 ± 7.8^∗∗^	113.6 ± 20.2^∗∗^
			(*n* = 7)
			Died in 10 h
			(*n* = 5)


*100 mg/kg of phenobarbital was intravenously injected to control and ALF mice. ALF, acute liver failure; PB, phenobarbital. Data are expressed as mean ± SD (*n* = 12). ^∗∗^*P* < 0.01 vs. control mice.*

### Effect of ALF on Phenobarbital Exposure in Mice Brain and Plasma

**FIGURE 4:**
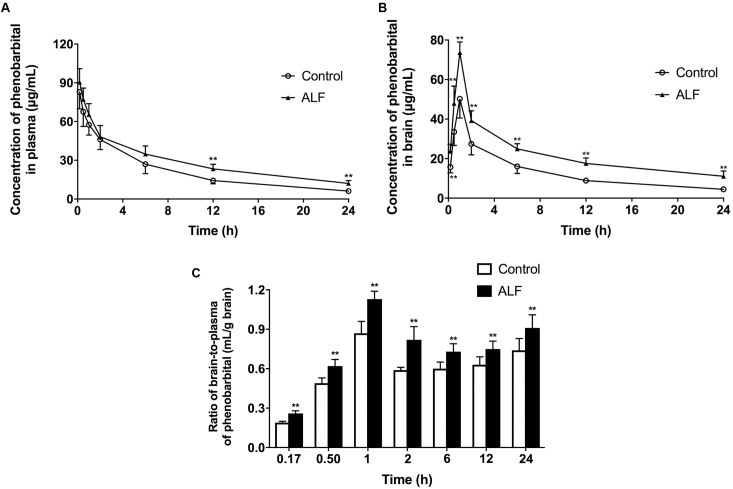
Mean plasma **(A)** and brain **(B)** concentration-time profiles, the brain-to-plasma ratios **(C)** of phenobarbital in ALF (filled triangle, shaded bar), and control (open circle, white bar) mice after i.v. administration of 60 mg/kg phenobarbital. The each point and bar represents the mean ± SD (*n* = 6), *P* < 0.01 vs. control mice.

The effect of ALF on phenobarbital concentrations in brain and plasma was studied. The data demonstrated that ALF not only increased plasma exposure (AUC_0-24 h_) of phenobarbital (680.5 ± 109.6 μg h/mL in ALF mice vs. 508.1 ± 84.3 μg h/mL in normal control mice, *P* < 0.05) (**Figure 4A**), but also significantly raised the brain distribution (AUC_0-24 h_) of phenobarbital (528.2 ± 68.4 μg h/g in ALF mice vs. 311.5 ± 42.4 μg h/g in control mice, *P* < 0.01) (**Figure 4B**). The brain-to-plasma concentration ratios of phenobarbital were calculated to reflect brain penetration (Jin et al., 2013). The results indicated that phenobarbital’s brain-to-plasma concentration ratio values in ALF mice were significantly greater than those in normal control mice (**Figure 4C**), which might have contributed to the enhanced sedative effect of phenobarbital in ALF mice.

### Effect of ALF on Activity and Expression of Hepatic Cyp3a11

**FIGURE 5:**
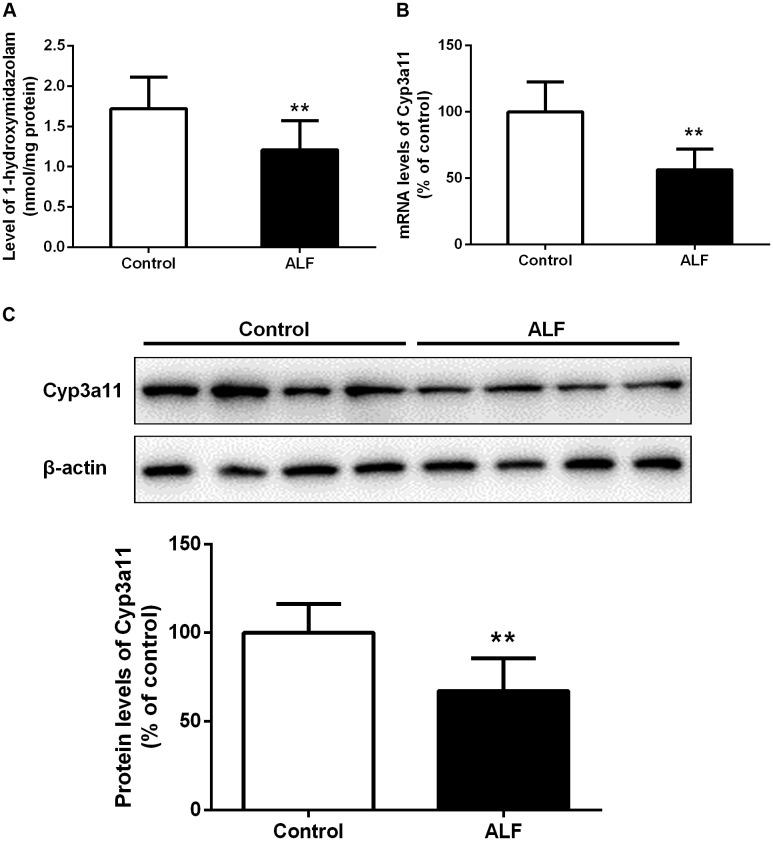
Formation of 1-hydroxylmidazolam from midazolam in hepatic microsomes of control (white bar) and ALF (shaded bar) mice **(A)**. The relative mRNA levels **(B)** and protein levels **(C)** of Cyp3a11 in hepatic microsomes of control (white bar) and ALF (shaded bar) mice. Data represent the mean ± SD (*n* = 6), ^∗^*P* < 0.05, ^∗∗^*P* < 0.01 vs. control mice.

Previous studies showed that phenobarbital is metabolized by hepatic CYP enzyme activity, predominantly by Cyp3a11 in mice (Chavan et al., 2015), inferring that the increased plasma concentration of phenobarbital could result from decreased activity of Cyp3a11 in ALF mice. To test this, we measured the activity of Cyp3a11 in liver microsomes of mice using 1-hydroxymidazolam formation from midazolam (**Figure 5A**). It was found that the formation of 1-hydroxymidazolam in hepatic microsomes of ALF mice was significantly lower than that in control mice. In line with this issue, significant decreases in mRNA and protein levels of Cyp3a11 in liver of ALF mice were observed (**Figures 5B,C**). The decreased mRNA and protein expression of Cyp3a11 in hepatic microsomes from ALF mice coincided with the diminished formation of 1-hydroxymidazolam, which indicated that the increased plasma exposure of phenobarbital might be partly due to the reduced activity and expression of Cyp3a11 in the liver of ALF mice.

## Discussion

The TAA-induced ALF mouse is a widely used ALF animal model, and might develop clinical signs that closely mimic the pathophysiological and clinical features of human ALF such as brain edema and alteration in neurological status (Faleiros et al., 2014). As expected, TAA-induced ALF mice were characterized with higher AST, ALT, ALP, TNF-α, and total bilirubin although serum ammonia level was unaltered. Higher MDA levels and lower GSH and CAT levels were also demonstrated in the brain of ALF mice, manifesting oxidative stress.

Our previous reports have demonstrated that liver failure altered the function and expression of P-GP, BCRP, and MRP2 at the BBB of rats (Jin et al., 2013; Zhang et al., 2014; Li et al., 2016). Findings in the BBB of ALF rats were confirmed in ALF mice. The expression and function of P-GP and BCRP were down-regulated, while those of MRP2 were up-regulated. Three typical substrates (rhodamine123, prazosin, and DNP-SG) of these transporters were used to evaluate the function of P-GP, BCRP, and MRP2 in the efflux system of the BBB *in vivo*, respectively. This was consistent with alterations in their expression compared to control mice, and the brain-to-plasma concentration ratios of rhodamine 123 and prazosin increased while the brain-to-plasma concentration ratio of DNP-SG decreased in ALF mice. Actually, since P-GP, BCRP, and MRP2 extensively overlap their tissue distribution, it is likely that these transporters considerably share pharmacological and toxicological functions, albeit with different (but partially overlapping) sets of substrates (Chaves et al., 2014). For example, MRP2-deficient TR rats demonstrated significant compensatory upregulation of P-GP on brain capillary endothelial cells in comparison with wild-type controls (Hoffmann and Löscher, 2007). The deficiency of P-GP in the mutant mdr1a^(-/-)^ mice was associated with increased BCRP (Abcg2) mRNA at the BBB (Cisternino et al., 2004). Similarly, the function and expression of P-GP and BCRP decreased, while the function and expression of MRP2 increased at the BBB of ALF mice in the study. It may be an important compensatory mechanism to protect the brain by limiting the entry of toxins.

The main findings in this study were that the function and expression of P-GP and BCRP decreased, while the function and expression of MRP2 increased in the brain of ALF mice. However, the mechanism by which these ABC transporter expressions were changed in TAA-induced ALF mice was unclear. We also reported that hyperammonemia enhanced the function and expression of P-GP and MRP2 at the BBB by activating the NF-κB pathway in a model of hyperammonemia-induced ALF rats (Zhang et al., 2014). This seemed contrary to the present study. This discrepancy may be partly determined by serum ammonia levels and there was no difference in serum ammonia levels between the ALF and normal control mice. Recently, we found that the elevated UCB may be a reason for impaired BCRP function and expression at the BBB of bile duct-ligated rats (Xu et al., 2016). Therefore, we subsequently investigated the effects of UCB and bile acids (CA, DCA, LCA, CDCA, and UDCA) on P-GP function and expression in HCMEC/D3 cells. The results showed that the uptake of two typical substrates of P-GP (rhodamine 123 and vincristine) was significantly increased in the presence of 100 μM CDCA in HCMEC/D3 cells. Meanwhile, the protein expression of P-GP was also down-regulated with 100 μM CDCA in HCMEC/D3 cells. However, the other tested bile acids had no effects on P-GP function in HCMEC/D3 cells. The findings were further validated by MDCK-MDR1 cells. All the above results suggested that CDCA, but not UCB, might be the pivotal serum component that attenuates P-GP function and expression at BBB of ALF mice.

To date, CDCA has been shown to increase MDR1 mRNA levels in renal epithelial cells (Kneuer et al., 2007). However, another study reported that CDCA did not affect the basal expression of P-GP in HepG2 cells, but antagonized a DOX-induced increase in P-GP expression (Komori et al., 2014). Here, we found that CDCA suppressed P-GP function and expression in HCMEC/D3 cells. These results indicate that the effect of CDCA on basal MDR1 gene expression may be dependent on the types of cells and tissues studied and thus remains to be further explored.

In addition to elevated bilirubin and total bile acids, ALF also resulted in increased circulating levels of proinflammatory cytokines including TNF-α, interleukin-1, and interleukin-6 (Bémeur and Butterworth, 2013). We found that the plasma TNF-α level was significantly increased in ALF mice. This proinflammatory cytokine might down regulate the expression and activity of P-GP and BCRP at the BBB according to previous studies (Poller et al., 2010; Iqbal et al., 2012). However, there were other investigations showing that proinflammatory cytokines could exert complex, dose- and time-dependent modulation of efflux drug transporters. For example, TNF-α showed biphasic effects on P-GP activity and expression in isolated rat brain capillaries, decreasing with a low dose, but increasing under longer exposure (6 h) (Pan et al., 2011; Miller, 2015).

Oxidative stress is another factor believed to play an important role in the pathogenesis of hepatic encephalopathy. Oxidative stress denotes an imbalance between production and neutralization of reactive oxygen species in the brain, which leads to cellular dysfunction and impairment of the BBB (Bosoi and Rose, 2013). Oxidative stress in the brain of ALF mice was indicated by remarkably increased MDA levels. Further, there were significantly declined GSH and CAT activities in the brain, although there was no difference in brain levels of NO in ALF and normal control mice. Oxidants/electrophiles are thought to increase the expression of ABC transporters (P-GP, BCRP, and MRP2) at the BBB (Miller, 2015) and GSH depletion upregulates P-GP expression at the BBB in rats (Wu et al., 2009). The above results imply that the altered function of P-GP, BCRP, and MRP2 might not only be due to the abnormal factors in serum and the imbalanced oxidative metabolism in brain of ALF mice, and the processes are more complicated and deserve further investigation.

In a physiological state, ABC efflux transporters form an active and selective barrier that protects the CNS by extruding metabolic wastes into the blood and limiting xenobiotics, like toxins, and a large number of drugs from entering into the brain (Hartz and Bauer, 2011). However, these transporters are affected by some diseases and expression alterations have been indicated as a prognostic factor in disease progression (Wanek et al., 2013). Furthermore, dysfunction of ABC transporters increased the exposure of potentially harmful concentrations of substances to the brain, disrupting brain homeostasis and neuronal signaling (Abbott et al., 2010). For example, ALF increased the neurotoxicity induced by cyclosporin A by lowering the P-GP function at the BBB (Yamauchi et al., 2011). Here, we examined whether the sedative effect of phenobarbital was altered under an ALF status and explored possible mechanisms by focusing on BBB permeability to phenobarbital.

Phenobarbital is widely used for treating insomnia and epilepsy, which acts mainly on γ-aminobutyric acid (GABA)/benzodiazepine receptors in the CNS. CNS depression is the main adverse effect of phenobarbital. A report showed that brain GABAergic tone rapidly increased along with the neuropathology of hepatic encephalopathy induced by acute or chronic liver failure (Sergeeva, 2013). This indicates that the increased sedative efficacy of phenobarbital in ALF mice might come from a raised GABAergic tone in the brain. However, the present study showed that ALF increases penetration of phenobarbital into the brain, which became a reason leading to the enhanced CNS activity of phenobarbital. Previous studies demonstrated that phenobarbital was a substrate of P-GP (Luna-Tortós et al., 2008; Zhang et al., 2012), but not for MRPs or BCRP (Luna-Tortós et al., 2010; Römermann et al., 2015). Data from MDCK-MDR1 and MDCK-BCRP cells also demonstrated that the transport of phenobarbital was regulated by P-GP but not BCRP. All results indicated that the attenuated expression and function of P-GP at the BBB might increase the uptake of brain phenobarbital and contribute to a longer duration of LORR and a shorter latency time in ALF mice.

Acute liver failure increased concentrations of phenobarbital in the plasma of mice. To elucidate the underlying mechanism of this phenomenon, hepatic Cyp3a11 activity and expression were evaluated because a report showed that phenobarbital is mainly metabolized by hepatic Cyp3a11 in mice (Chavan et al., 2015). This was consistent with our expectation that the activity and mRNA expression of hepatic Cyp3a11 would decrease. Other hepatic Cyp450s including Cyp1a2, Cyp2b1/2, Cyp2c6, and Cyp2e1 also down-regulated when there was liver injury (Xie et al., 2013, 2014). All of these alterations in hepatic Cyp450s might partly contribute to the elevated plasma exposure of phenobarbital in ALF mice. Thus, the net penetration into the brain of phenobarbital was further calculated as brain-to-plasma concentration ratios in order to assess the contribution of increased plasma concentrations of phenobarbital to sedation on CNS under ALF. The results demonstrated that the brain net penetration of phenobarbital in ALF mice was always significantly higher than control mice after intravenous administration of phenobarbital, which might partly lead to the enhanced sedative effect of phenobarbital in ALF mice, even though the plasma exposure of phenobarbital was also raised in ALF mice.

The regulation of BBB integrity is a complicated result of many factors when exposed to an ALF condition. Accumulating evidence has demonstrated that the BBB has only minimal ultrastructural changes in the brain capillaries of animals and humans with ALF (Scott et al., 2013). Nguyen indicated that BBB dysfunction with ALF is associated with protein deregulation in tight junctions (TJs) and increased permeability only to small molecules like water and ammonia but is not necessarily associated with a structural breakdown (Nguyen, 2012). Although there has been little evidence of a complete BBB breakdown, findings from more recent studies suggest that matrix metalloproteinase-9 (MMP-9), a member of the MMP family of endopeptidase enzymes, causes protein degradation of TJ and is upregulated in the liver of ALF mice (Chen et al., 2013; McMillin et al., 2015). Additionally, increased inflammatory cytokines including interleukin-1 (IL-1), IL-6, and TNF-α, which are released from the breakdown products of injured and necrotic hepatocytes, were responsible for TJ protein downregulation through TLR4/NF-κB signaling and also resulted in an apparent increase in BBB permeability (Aslam et al., 2012). Taken together, these findings suggest that substances derived from the injured liver, such as MMP-9 and TNF-α, reach the BBB and induce increased permeability through subtle changes in TJ composition (Scott et al., 2013).

The elevated brain concentrations of phenobarbital and substrates of P-GP might result from increased permeability at the BBB of ALF mice. Therefore, a permeable experiment of Evans blue was conducted. Evans blue binds strongly to albumin, forming a high-molecular complex that does not readily penetrate the BBB. The results found a slight extravasation of Evans blue, and this was not significantly increased in the brain of ALF mice. This observation coincided with a previous report (Li et al., 2016). Based on these findings, it is possible that BBB permeability was either normal or tended to increase under ALF status.

## Conclusion

Our study confirmed that the function and expression of P-GP and BCRP decreased, while the function and expression of MRP2 increased in the brain of ALF mice. The attenuated function and expression of P-GP at the BBB might enhance the distribution of phenobarbital in the brain, and subsequently increase its efficacy and toxicity on CNS in ALF mice. Thus, it appears necessary to adjust the dosage of phenobarbital medicines in patients with liver diseases.

## Author Contributions

LL and XL participated in the research design. LL, YC, ZW, BS, and MM conducted the experiments. LL, XL, and ZW performed the data analysis. LL and XL wrote the manuscript.

## Conflict of Interest Statement

The authors declare that the research was conducted in the absence of any commercial or financial relationships that could be construed as a potential conflict of interest.
